# Metabolic Syndrome: Epidemiology, Pathophysiology, and Nutrition Intervention

**DOI:** 10.1155/2012/584541

**Published:** 2012-06-07

**Authors:** Isaias Dichi, Andréa Name Colado Simão, Hélio Vannucchi, Rui Curi, Philip C. Calder

**Affiliations:** ^1^Department of Internal Medicine, University of Londrina, 86038-440 Londrina, PR, Brazil; ^2^Department of Pathology and Clinical Analysis, University of Londrina, 86038-440 Londrina, PR, Brazil; ^3^Department of Internal Medicine and Clinical Nutrition, University of São Paulo (USP), 14049-900 Ribeirão Preto, SP, Brazil; ^4^Department of Biomedical Sciences and Physiology, University of São Paulo (USP), São Paulo, 14049-900 Ribeirão Preto, SP, Brazil; ^5^School of Medicine, University of Southampton, IDS Building, MP887 Southampton General Hospital, Tremona Road, Southampton SO16 6YD, UK

Metabolic syndrome (MetS) comprises pathological conditions that include insulin resistance, arterial hypertension, visceral adiposity, and dyslipidemia, which favor the development of cardiovascular (CV) diseases. MetS is rising in developed and in developing countries, in male and female subjects, and in adults, adolescents, and children. Although obesity and insulin resistance comprise the leading causes of MetS, many other pathophysiological mechanisms, like proinflammatory adipocitokines, hyperuricemia, nitric oxide, and oxidative stress may contribute to the potential cardiovascular risk factors related to the syndrome. On the other hand, increases in anti-inflammatory adipocitokine, as adiponectin, and antioxidant mechanisms could give some CV protection. Nutritional intervention has been attempted to attenuate the detrimental or increase the beneficial mechanisms in order to decrease the potential risk of hypertension, glucose intolerance and diabetes, lipid disorders, and abdominal obesity, which are the main clinical features of MetS. 

In the present special issue on MetS, original research articles as well as review articles contribute to the continuing efforts to understand the pathophysiology underlying MetS and the development of strategies to treat these conditions through nutrition intervention. 

Several aspects of pathophysiology and nutrition intervention will be found in this special issue on MetS. Four articles evaluate the role of inflammation and adipocytokines on the syndrome. T. Di Chiara et al. have proposed in their review that hypoadiponectinemia is the most interesting new hypothesis to explain the pathophysiology of MetS; the review by Emanuela et al. also proposes inflammatory status as a link between obesity and MetS; L. Pala et al. in a case-control study assessed the relationship among several adipokines (adiponectin, retinol-binding protein 4, adipocyte fatty acid binding protein, and visfatin) and incident CV diseases, and A. N. C. Simão et al. evaluated several markers related to MetS and their association to adiponectin levels. All together, these papers reinforce the suggestion that hypoadiponectinemia may exert a fundamental role in the passage from visceral obesity to MetS. 

The review article from H. La Guardia et al. examines the link between MetS and chronic kidney disease, exploring both pathophysiology and intervention strategies. 

Nutritional intervention is also evaluated in A. Branchi's et al. and M. Waling's et al. studies. In the former, postprandial changes in serum lipoproteins and blood glucose were evaluated in two different mixed meals (low-fat or low-carbohydrate diets). In the later, the effects after one-year family-based intervention were assessed on anthropometrics and metabolic markers in overweight and obese children.

*Isaias Dichi* *Isaias Dichi* *Andréa Name Colado Simão* *Andréa Name Colado Simão* *Hélio Vannucchi* *Hélio Vannucchi* *Rui Curi* *Rui Curi* *Philip C. Calder* *Philip C. Calder*

